# Accumulating Variation at Conserved Sites in Potyvirus Genomes Is Driven by Species Discovery and Affects Degenerate Primer Design

**DOI:** 10.1371/journal.pone.0001586

**Published:** 2008-02-13

**Authors:** Linda Zheng, Paul J. Wayper, Adrian J. Gibbs, Mathieu Fourment, Brendan C. Rodoni, Mark J. Gibbs

**Affiliations:** 1 School of Botany and Zoology, Australian National University, Canberra, Australian Capital Territory, Australia; 2 Yarralumla, Australian Capital Territory, Australia; 3 Department of Biological Sciences, Macquarie University, Sydney, New South Wales, Australia; 4 Department of Primary Industries, Knoxfield, Victoria, Australia; National Institute for Communicable Diseases, South Africa

## Abstract

Unknown and foreign viruses can be detected using degenerate primers targeted at conserved sites in the known viral gene sequences. Conserved sites are found by comparing sequences and so the usefulness of a set of primers depends crucially on how well the known sequences represent the target group including unknown sequences.

**Methodology/Principal Findings:**

We developed a method for assessing the apparent stability of consensus sequences at sites over time using deposition dates from Genbank. We tested the method using 17 conserved sites in potyvirus genomes. The accumulation of knowledge of sequence variants over 20 years caused ‘consensus decay’ of the sites. Rates of decay were rapid at all sites but varied widely and as a result, the ranking of the most conserved sites changed. The discovery and reporting of sequences from previously unknown and distinct species, rather than from strains of known species, dominated the decay, indicating it was largely a sampling effect related to the progressive discovery of species, and recent virus mutation was probably only a minor contributing factor.

**Conclusion/Significance:**

We showed that in the past, the sampling bias has misled the choice of the most conserved target sites for genus specific degenerate primers. The history of sequence discoveries indicates primer designs should be updated regularly and provides an additional dimension for improving the design of degenerate primers.

## Introduction

About 55% of known virus species have linear single-stranded RNA genomes; potyviruses belong in this category. RNA virus genomes are diverse in plan and variable in sequence [1]–[4]. Different isolates from the same species usually have many single nucleotide polymorphisms. All RNA viruses have RNA dependent RNA polymerase (RdRp) genes, but even though they are the most conserved genes, they are very varied [5]. Therefore to identify RNA virus species, degenerate primers (i.e. primer mixtures) that target conserved sites have been developed for many viral genera. For some important genera, several sets of degenerate primers have been designed. Conserved sites are found by comparing sequences and so the usefulness of a set of primers depends crucially on how well the available sequences represent the target viruses, including as yet unknown members of the genus. At any one time, the available sequences only represent a sample of the extant and future target sequences, so biases in this sample can affect degenerate primer design. Understanding such biases is important, as degenerate primers are an essential biosecurity technology allowing viruses and microbes to be detected and identified and because they are essential for research on virus diversity, ecology and phylogeny [6], [7].

The genus *Potyvirus* is the second largest known virus genus, with 111 recognised species and 86 tentative species currently assigned to it by the International Committee on Taxonomy of Viruses (ICTV)[3]. Potyviruses infect plants and cause serious diseases in crops worldwide [6], [7]. By 2006 the international gene sequence databases contained more than 250 fully sequenced potyvirus genomes, representing 47 species. This large sample of complete genomes of different species is second only to that of the genus *Begomovirus.* Extensive studies of potyvirus sequences have shown that potyvirus species can be distinguished by sequence comparisons, and the process of recognising a species is now in large part determined using this measure. Potyvirus isolates with 85% sequence identity or more over the whole genome are usually considered to be from the same species [3]. This was re-evaluated by Adams *et al*. in 2005, who suggested that a value of about 76% was most appropriate for the entire polyprotein sequence and that slightly different values might be used for the different regions of the potyvirus genome [8]. We have chosen to use the value adopted by the ICTV.

Since the early 1990s, six sets of primers have been developed and used widely for potyviruses; the sites are commonly named after the amino acid motifs that they encode. Nicholas and Laliberte published the first degenerate primers in 1991, which targeted the ‘ATNIIENG’ and ‘YCDADGS’ sites in the cylindrical cytoplasmic inclusion protein (CI) and RdRp (NIb) genes [9]. Only five potyvirus sequences were available to Nicholas and Laliberte and they based their designs on conserved amino acids. Four more sequences were used by Langeveld *et al*. in the same year to design primers to the AHFQMKTA and TVVDNTLMV sites in the NIb gene and the MVWCIENG site in the coat protein (CP) gene [10]. Working with 15 sequences in 1993, Pappu *et al*. produced the WCIEN primer, which targeted a subsequence from the MVWCIENG region, and the QMKAAA primer also from the CP gene [11]. These degenerate primers were paired with an oligo-d(T) primer that annealed to the poly-adenylate tail that is present at 3′ end of all potyvirus genomes. In the same year, Colinet and Kummert reported the QMKAAA primer independently, and produced primers to the WKHWI and WNGSL sites from the NIa-Pro and NIb genes [12]. They also used a primer to the CDADGS site identified earlier. Four years later, Gibbs and Mackenzie reported a primer that matched the GNNSGQ motif of the RdRp and found that the primer could be used to amplify cDNA from viruses from other genera in the family *Potyviridae* as well as from potyviruses [13]. In 2001, Chen *et al*. published an extended version of the GNNSGQ primer which included a few more nucleotides [14].

We have developed a method for analysing the apparent stability of conserved sites as more sequences are discovered and reported. Here we report an analysis by this method of conserved sites in potyvirus genomes, including some of those previously listed. We analysed the sequences reported over a 20 year period and showed how knowledge of nucleotide variants accumulates as the sequence database builds. The variants produced an apparent decay of the consensus at the sites. We found that the decay rates differed at different sites and hence the ranking of sites by their conservation changed over time. To help interpret this data, we cross-checked lists of the species names declared in the GenBank and ICTV databases and tested the sequence-based definitions of the species to track the rise in the numbers of known species and analysed datasets from the three most fully sampled species. We compared the consensus decay rates within and between species in order to assess whether variation among isolates from single virus species contributed to the decay. Our results suggest that measuring consensus decay at conserved sites is important when designing primers for detecting both known and unknown viruses.

## Results

Using all the available full length sequences, we found 13 conserved sites that had not been previously reported (Table 1). Seven of the sites were found by eye and six were found using the NCSF program (see Materials and Methods). Four of the eight sites identified by earlier workers were included: GNNSGQ, MVWCIEN , NIIENG and QMKAAA [9]–[11], [13], [14]; the 5′ nucleotide of the regions encoding these motifs were at positions 7899, 8853, 4545 and 9162 in the *Tobacco etch virus* genome (Accession code M11458). The other four sites that had been reported earlier were found to be very variable and were not included, although parts of two of them, TVVDNTLMV and YCDADGS [10], overlapped with two of the newly identified sites. Five of the 17 sites selected for analysis (sites 3, 4, 7, 11 and 15; Table 1) were clustered in the CI gene and the remainder of the sites were located in the NIb and the CP genes (Table 1). This clustering was consistent with the reported variability along potyvirus genomes [15]. None of the newly recognised sites encoded motifs associated with known protein functions.

**Table 1:** Rank and N scores of 17 conserved sites in potyvirus genomes along with their locations and sequence and the method used to identify each one.

rank^1^	location^2^	Gene/ORF^3^	nucleotide sequence of sites (5′—3′)	amino acid sequence^4^	identified by^5^	N scores
1	7587	NIb	TGYGTNGAYGAYYTYAAYAA	CVD*DFN*	Eye/E1/R1/C2/V3	0.55
2	9237	CP	GARRAYACDGARMGNCAYRC	EN/D*T*ERH	R4/C5/V1(9240)^6^	0.60
3	4545	CI	RAYATHRTNGARAAYGGNGT	*N*I*I*ENGV	Nicholas^7^/E5	0.65
4	4539	CI	GCNMSNRAYATHRTNGARAAYGG	A*TN*I*I*ENG	Eye	0.78
5	9162	CP	CARATRAARRCNKCNSV^8^	QMKAAA	Langeveld/Pappu^9^	0.82
6	8278	NIb	SNATNDTNGADKCNTGGGG	*A/SMI/VE*S/AWG	Eye	0.84
7	4458	CI	AARRTNGAYGGNMGNWCNAT	KV/IDGRT/SM	R5	0.85
7	7899	NIb	GKNAAYAAYWSBGGNCARCC	*G*NNSG*Q*	Gibbs^7^/R3	0.85
8	9099	CP	WWHGSNTTYGAYTTHTWHVR	*YAF*D*FYE*	E3/C3	0.90
9	7911	NIb	GGNCARCCNTCNACNGTNGTNG	G*Q*P*S*TVV*D*	Eye	0.95
10	7545	NIb	TTYACNGCNGCNCCNNTNGRNAC	FTAAP*L/ID/E*	Eye	1.00
10	7722	NIb	GAYGGNWSNMRVYTYGAYWS	DG*S*Q/R*FDS*	E4	1.00
11	3888	CI	GTNGGNWSNGGNAARTSNWC	*V*GSGK*ST*	Eye	1.05
12	8853	CP	WKGRTNHGGKNHHTNGDNAR	*MVWC*I*E/DN*G	C1/E2/V4/Langeveld/Pappu^10^	1.10
13	8901	CP	TGGNHNWTSRTRVAHRRNVR	W*V/TMMDG*D/*E/N*	C4/V5	1.20
13	9042	CP	NNNTAYATDSCNVGNTRYGS	*P/R/AYMP*R*YG*	V2	1.20
14	4396	CP	CNRGYYRYRRHGANRTNGA	*A/SSYN*E/D*V*D	Eye	1.21

1 Sites with equal N scores (see Materials and Methods) were ranked as equal (e.g. 7th for sites at position 4458 and 7899)

2 Nucleotide positions refer to the 5′ residue of the site in the sequence of the *Tobacco etch virus* genome [Accession code M11458].

3 CI (cylindrical inclusion), a multifunctional protein that acts as an RNA helicase and is involved in genome replication and cell-to-cell movement; NIb (large nuclear inclusion protein), RdRp (RNA-dependent RNA polymerase); CP (coat protein), RNA encapsidation.

4 Only the most abundant variants, those present in 8 or more potyvirus genomes are shown. Italicised amino acids have other variants present at the same site in fewer than 8 potyvirus genomes.

5 Regions were identified by using conservation measures implemented in the program NCSF are coded C1 to C5, E1 to E5, R1 to R5, or V1 to V5 where the codes represent the ranking according to the dominant base count(C), Shannon entropy score (E), redundancy score (R) or sub-sequence variants count (V).

6 R4 and C5 both start from 9237 whereas V1 starts from 9240

7 Nicholas and Laliberte (1991) identified part of this motif and designed primers that targeted NIIENG. This is part of a conserved region 69 nucleotides long (with one nucleotide gap) that encodes: KKH/FKG/VNNSGQPSTVVDNTLMVV/II. Langeveld *et al.* (1991) identified part of the region and designed primers that targeted TVVDNTLMV. Gibbs and Mackenzie (1997) designed primers that targeted G/VNNSGQ in this region.

8 One sequence (Accession code AJ310102) was removed from analysis of this site as there was 1 codon (3 nucleotides) insertion at position 9165.

9 Langeveld *et al.* (1991) identified part of this motif and designed primers that targeted AHFQMKTA. Pappu *et al.* (1993) modified Langeveld's primer and designed primer that targeted QMKAAA.

10 Langeveld *et al.* (1991) identified this motif and designed primers to target MVCIENG. Pappu *et al.* (1993) designed primers to target WCIEN.

We ranked the 17 sites according to their level of conservation as measured by the average nucleotide variant count (see Materials and Methods) using the sequences available up to December 2005 (Table 1). The CVD*DFN* site at position 7587 in the NIb gene was the most conserved site, followed by the EN/D*T*ERH site at position 9237 in the CP gene. The CVD*DFN* site was identified by eye, and had the smallest Shannon entropy and redundancy scores. It also had small dominant base and sub-sequence variant counts. The EN/DTERH site was found using NCSF and had the smallest sub-sequence variant count. It also had a small redundancy score and a small dominant base count. Of the previously identified sites, NI*I*ENGV found by Nicholas and Laliberte (“ATNIIENG”) [9] ranked third, QMKAAA found by Langeveld *et al.* [10] and Pappu [11] ranked fifth, GNNSGQ site identified by Gibbs and Mackenzie (PV2) [13] ranked equal seventh, and the MVWCIE/DNG site identified by both Langeveld *et al.* [10] and Pappu (“WCIEN”) [11] ranked 12^th^. Three of those published sites were found by NCSF to be among the top five sites ranked on one or other of its 4 measures. The WVCIEN site was not among the top five scores in any of the NCSF measures.

### Sequences and Species

Only 90 (81.1%) of the 111 confirmed potyvirus species and 11 (12.8%) of the 86 tentative species recognised by the ICTV were represented by sequences recorded in GenBank. We assume that no sequence data has been reported for the remaining 21 recognised and 75 tentative species. The value of recognising potyvirus species without sequence data is uncertain. We found 64 sequences from potyviruses with names that had not been listed by the ICTV. Our database searches suggested that 25 of those unrecognised sequences probably came from distinct unrecognised species, 35 had been misnamed as they matched the sequences of recognised species, and 4 were difficult to categorise because they differ from the sequences of recognised species but not conclusively so (data not shown).

**Figure 1:**
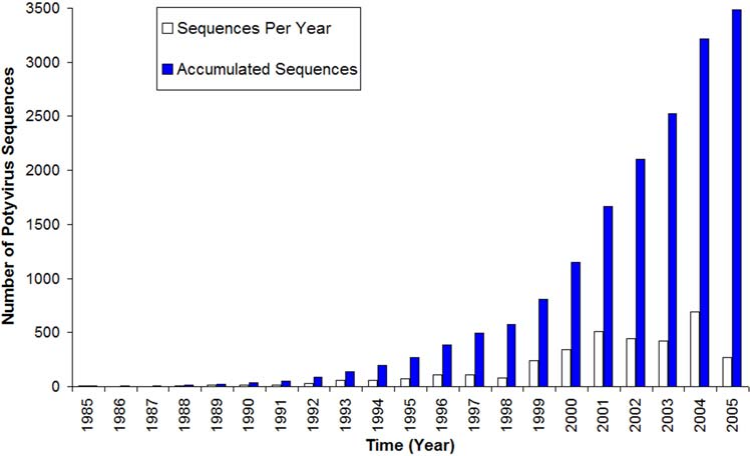
The annual release and the accumulated total of all potyvirus sequences in GenBank from 1985 to 2005.

**Figure 2:**
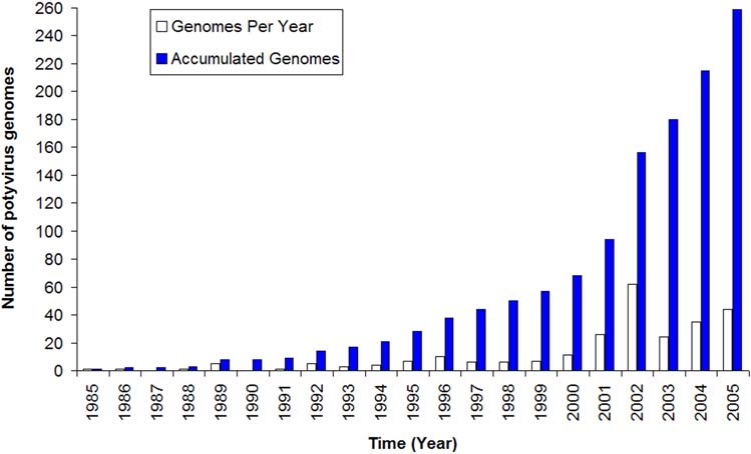
The annual release and the accumulated total of all full length potyvirus sequences in GenBank from 1985 to 2005.

The numbers of full length and partial sequences submitted to Genbank annually from 1985 to 2005 were plotted, together with the cumulative total number of sequences (Figure 1). Very few new sequences were reported between 1985 and 1992, but the rate increased after that period and peaked between 2001 and 2005 with an overall mean of 165.9 sequences/year. The number of full length sequences released each year followed a similar trend with a peak of more than 50 full length sequences being deposited in 2002 (Figure 2; mean 12.3). Interestingly, the number of named species associated with gene sequences submitted to Genbank differed markedly from the number of species recognised by the ICTV each year (Figure S1; mean 4.2) and neither showed any clear trend. The yearly count of potyvirus species found to have distinct nucleotide sequences varied considerably and no clear trends was observed (Figure S2), but on average 5.8 new species were identified annually by this means. The peak years for species officially recognized by the ICTV were 1999 and 2002, and 2004 was the peak year for new species included in GenBank (Figure S2).

### Accumulating nucleotide variants

**Figure 3:**
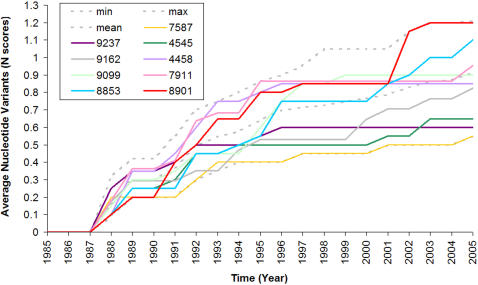
Average nucleotide variant score (N score) of the top 3 and 6 other conserved sites compared with the minimum, maximum and average N scores of all 17 conserved sites in the potyvirus genomes.

A potyvirus genome was completely sequenced for the first time in 1985, and nucleotide variants were first recorded in 1988, so we largely confined our analyses to the years after 1988. Average nucleotide variant counts (N scores) were calculated for each of the 17 primer sites for each year after 1988 using the data from the genome dataset. N scores increased with time for all sites including the most conserved (sites 7587, 9237 and 4545; Figure 3). N scores also diverged over time as shown by the standard deviations (Table 2); the SD value for the conserved sites rose from 0.07 to 0.2 over the 17 years from 1988. Randomly selected sites accumulated nucleotide variants much more rapidly with SD values double those found for the conserved sites (Table 2). The mean N score in 2005 was 2.02 for the randomly selected sites (Table 2), whereas the best and worst conserved sites had scores of 0.55 and 1.20 (Table 1).

**Table 2:** Average nucleotide variant counts (N scores) of 17 conserved sites compared to the mean of 20 random sites in all potyvirus genomes from 1985 to 2005.

conserved sites	85	86	87	88	89	90	91	92	93	94	95	Yr 96	97	98	99	00	01	02	03	04	05
**7587**	0.00	0.00	0.00	0.20	0.20	0.20	0.20	0.30	0.40	0.40	0.40	0.40	0.45	0.45	0.45	0.45	0.50	0.50	0.50	0.50	0.55
**9237**	0.00	0.00	0.00	0.25	0.35	0.35	0.40	0.50	0.50	0.50	0.55	0.60	0.60	0.60	0.60	0.60	0.60	0.60	0.60	0.60	0.60
**4545**	0.00	0.00	0.00	0.10	0.25	0.25	0.30	0.45	0.45	0.50	0.50	0.50	0.50	0.50	0.50	0.50	0.55	0.55	0.65	0.65	0.65
**4539**	0.00	0.00	0.00	0.09	0.26	0.26	0.30	0.43	0.43	0.43	0.57	0.57	0.57	0.57	0.61	0.65	0.70	0.70	0.78	0.78	0.78
**9162**	0.00	0.00	0.00	0.18	0.29	0.29	0.29	0.35	0.35	0.47	0.53	0.53	0.53	0.53	0.53	0.65	0.71	0.71	0.76	0.76	0.82
**8278**	0.00	0.00	0.00	0.32	0.42	0.42	0.47	0.53	0.68	0.74	0.74	0.74	0.74	0.74	0.74	0.74	0.79	0.84	0.84	0.84	0.84
**4458**	0.00	0.00	0.00	0.10	0.35	0.35	0.45	0.60	0.75	0.75	0.80	0.85	0.85	0.85	0.85	0.85	0.85	0.85	0.85	0.85	0.85
**7899**	0.00	0.00	0.00	0.20	0.30	0.30	0.35	0.45	0.45	0.45	0.50	0.55	0.55	0.55	0.70	0.70	0.70	0.75	0.75	0.75	0.85
**9099**	0.00	0.00	0.00	0.15	0.30	0.30	0.35	0.45	0.45	0.45	0.60	0.75	0.85	0.85	0.90	0.90	0.90	0.90	0.90	0.90	0.90
**7911**	0.00	0.00	0.00	0.18	0.36	0.36	0.41	0.64	0.68	0.68	0.86	0.86	0.86	0.86	0.86	0.86	0.86	0.86	0.86	0.86	0.95
**7545**	0.00	0.00	0.00	0.09	0.30	0.30	0.35	0.48	0.70	0.74	0.83	0.87	0.87	0.91	0.91	0.96	0.96	0.96	1.00	1.00	1.00
**7722**	0.00	0.00	0.00	0.25	0.30	0.30	0.40	0.45	0.50	0.50	0.60	0.65	0.70	0.70	0.80	0.85	0.85	0.85	0.85	0.90	1.00
**3888**	0.00	0.00	0.00	0.15	0.40	0.40	0.50	0.65	0.70	0.70	0.75	0.90	0.90	0.90	0.90	0.95	0.95	1.00	1.00	1.00	1.05
**8853**	0.00	0.00	0.00	0.10	0.25	0.25	0.25	0.45	0.45	0.50	0.55	0.75	0.75	0.75	0.75	0.75	0.85	0.90	1.00	1.00	1.10
**8901**	0.00	0.00	0.00	0.10	0.20	0.20	0.40	0.50	0.65	0.65	0.80	0.80	0.85	0.85	0.85	0.85	0.85	1.15	1.20	1.20	1.20
**9042**	0.00	0.00	0.00	0.20	0.30	0.30	0.55	0.70	0.70	0.80	0.80	0.90	0.95	1.05	1.05	1.05	1.05	1.15	1.15	1.15	1.20
**4396**	0.00	0.00	0.00	0.11	0.26	0.26	0.32	0.47	0.47	0.53	0.58	0.63	0.63	0.68	0.74	0.74	0.79	0.84	0.95	1.11	1.21
**Mean**	**0.00**	**0.00**	**0.00**	**0.16**	**0.30**	**0.30**	**0.37**	**0.49**	**0.55**	**0.58**	**0.64**	**0.70**	**0.71**	**0.73**	**0.75**	**0.77**	**0.79**	**0.83**	**0.86**	**0.87**	**0.92**
**SD**	**0.00**	**0.00**	**0.00**	**0.07**	**0.06**	**0.06**	**0.09**	**0.10**	**0.13**	**0.13**	**0.14**	**0.16**	**0.16**	**0.17**	**0.17**	**0.16**	**0.15**	**0.18**	**0.18**	**0.19**	**0.20**
**random sites mean**	**0.00**	**0.00**	**0.00**	**0.41**	**0.88**	**0.88**	**1.05**	**1.29**	**1.41**	**1.46**	**1.59**	**1.69**	**1.72**	**1.74**	**1.78**	**1.81**	**1.85**	**1.91**	**1.94**	**1.98**	**2.02**
**random sites SD**	**0.00**	**0.00**	**0.00**	**0.16**	**0.22**	**0.22**	**0.25**	**0.30**	**0.34**	**0.34**	**0.35**	**0.39**	**0.39**	**0.40**	**0.41**	**0.40**	**0.40**	**0.43**	**0.41**	**0.42**	**0.42**

When sites were ranked using N scores, the rankings changed over time. Site 8901 was ranked equal best in 1989 and 1990, but was ranked worst in 2003 and 2004 (Table 2). Site 8853 was ranked second best in 1989–1991, but was ranked third worst in 2005 (Table 2; Figure 3). Conversely, site 9237 was ranked second worst in 1988, but became the second best site by 2003 and remained in that position through to 2005 (Table 2). By contrast, some sites had a stable ranking; site 7587 (CVDDFN) was the most conserved site over almost all years after 1988 (Table 2; Figure 3).

The consensus decay of some sites appeared to stabilize after a time, as their N scores asymptoted. For example, no further nucleotide variants were reported after 1996 at sites 4458 and 9237, and no consensus decay occurred at site 9099 after 1999. Site 7911 was stable for nine years, but some more variants of that site were reported in 2005. The mean yearly N score of all conserved sites climbed in all years except 1990; there were obvious pauses and jumps in this value in the early years and more steady increases after 1995. The overall rate of change of the N scores between 1988 and 2005 was 0.044 variants per position per year (v/p/y) as calculated from the slope of the mean of yearly N scores of all conserved sites. The slope of the mean of all conserved sites between 1988 and 1995 was 0.06 v/p/y, and between 1996 and 2005 it was 0.024 v/p/y.

The relationship between variability and the accumulated number of sequences was calculated by dividing the mean of the N scores of the conserved sites by the number of sequences (Figure S3). A peak value (0.0147) was observed in the first year that variants were observed (1988) and after that year, the value rapidly approached zero, indicating that as the total number of the sequences increased, fewer variants per sequence were observed (Figure S3).

**Figure 4:**
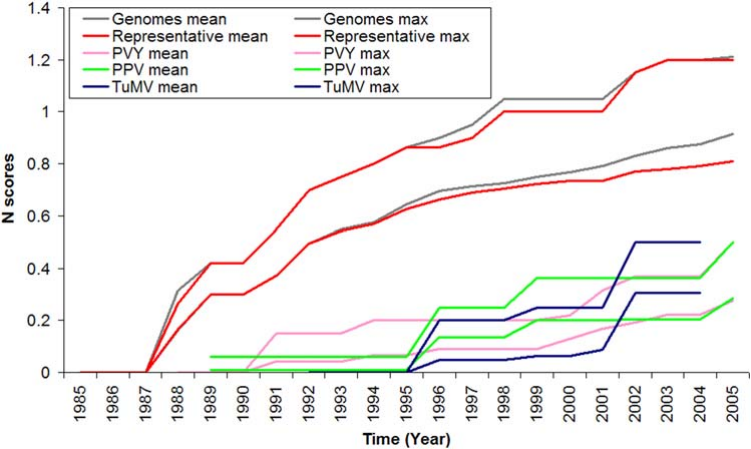
Comparison of the maximum and mean N scores for all conserved sites in the genome, representative and the species datasets.

To estimate the differing contributions from species and strains, i.e. the within and between-species variation, we calculated N scores for the three species datasets and the representative dataset. The N scores from the representative dataset were about the same, from year to year, as those from the genome dataset (Table S1), with small differences apparent after 1995 (Figure 4). The N scores from the species datasets revealed more information as they were small or zero before 1995 and rose sharply after that year (Figure 4), probably because few sequences were obtained from the three species in early years while many sequences were obtained in the last few years. N scores from the PPV, TuMV and PVY datasets had mean rates of change of 0.013 v/p/y, 0.039 v/p/y and 0.022 v/p/y respectively. The differences between the genome and representative datasets, visible after 1995 (Figure 4), were probably due to sequences from strains. They could be interpreted to show a small bias in the results from the genome dataset due to over-representation of some species, i.e. those represented by many strain sequences. As expected, N scores from the genome dataset correlated much more strongly with those from the representative dataset than with those from the species datasets (Table 3).

**Table 3:** Correlations between the N score time series from the genome dataset and the N score time series from other datasets for each site.

	correlation coefficients between datasets			
conserved sites	representative	PPV	TuMV	PVY
**4396**	0.98	0.90	0.89	0.88
**9042**	1.00	N/A	0.65	0.58
**8901**	1.00	0.80	0.86	0.92
**8853**	0.98	0.92	0.87	0.62
**3888**	1.00	0.86	0.80	0.80
**7722**	1.00	0.92	0.67	0.83
**7545**	1.00	0.80	0.58	0.73
**7911**	1.00	0.76	0.33	0.65
**9099**	1.00	0.89	0.61	0.50
**7899**	0.99	0.93	0.71	0.93
**4458**	1.00	0.75	0.78	0.52
**8278**	0.99	0.76	0.71	0.92
**9162**	0.98	0.42	0.72	0.84
**4539**	0.98	0.89	0.89	0.92
**4545**	0.97	0.76	0.89	0.84
**9237**	1.00	0.84	0.35	0.80
**7587**	0.99	0.83	0.72	0.91
**average**	**0.99**	**0.81**	**0.71**	**0.78**
**minimum**	**0.97**	**0.42**	**0.33**	**0.50**
**maximum**	**1.00**	**0.93**	**0.89**	**0.93**

* N/A–no variants occurred at this site in the PPV genomes

## Discussion

Consensus decay is important, as degenerate primer mixtures only reliably detect all the target viruses if they contain individual primers that complement and bind to all the virus sequences. We found that several of the sites targeted in early work on potyviruses were no longer conserved. This is likely to be a general effect as, in the first few years after the first genomes of a new genus are sequenced, a consensus sequence will not cover many of the sequence variants.

Consensus decay may mislead the degenerate primer design process. We have shown that conserved sites in potyvirus genomes have been incorrectly ranked in the past. The rank of every one of the conserved sites targeted by earlier workers changed over time (Table 2), and the most conserved of those sites was only ranked third in 2005 (Table 1). We found that all conserved sites in potyvirus genomes suffer consensus decay (Table 2), however different sites asymptote to different levels of variability. For example, sites 9237 and 4458 both attained a stable variant count by 1996, however the first had an average variant count of 0.6 and the latter of 0.85 (Table 2; Figure 3).

Consensus decay across sites from a genus is largely due to variation between species, rather than variation within a species, as was shown by the correlation between the N scores from the genome and representative datasets (Table 3; Figure 4). New strain sequences are being generated continually by mutations [2], [4], [16], but this may not have a significant effect on the N score of the genus. Many nucleotide variants were reported after 1995 among the strains of PPV, PVY and TuMV, but they had little effect on the consensus decay of the genome dataset, presumably because the same variants had already been recorded. If the strain variants had been novel we would have expected N scores to have increased at a higher rate after 1995. We suggest that the virus species analysed in this study come from lineages that have explored a large proportion of the viable sequence space [17], and although mutation continues within each species, it is largely a reiteration of earlier exploration. Hence, additional sampling of strains is mostly finding variants that are already known.

We counted the number of potyvirus species confirmed by sequence deposition each year, and found an average rate of 5.8 per year, although the totals varied greatly (Figure S2). Likewise, peaks in the number of new species recognised by the ICTV varied greatly. These variations probably reflect irregular sampling. Many human factors probably contributed to the species sampling variability including motivation, funding and improvements in molecular methods and primer design. Early sequencing efforts appear to have concentrated on the well known virus species that caused significant crop damage, whereas later efforts have been to identify less damaging species. There are inconsistencies between species and sequence sampling. In the past recognition and discovery of a species has often preceded sequencing, whereas more recently species recognition has lagged behind sequencing and species discovery. The peak years of species discovery may have past and few new potyvirus species have been recognised by the ICTV since 2002 (Figure S2). However, the year to year counts are very variable (Figures S1 and S2), so much is uncertain. It should also be realised that few wild plants have yet been tested for potyviruses, so there may be many new species still to be discovered and further consensus decay may occur.

In conclusion, we believe consensus decay will be measured in the future when new degenerate primers are designed or existing ones are updated. Quarantine agencies and authorities involved in biosecurity may use consensus decay data when assessing degenerate primer or probe tests. Other factors will be considered, first of which will be empirical data on primer performance, and second may be the location of the primers in the genome and the proximity of their target sites. It is worth repeating that degenerate primers designed in the past for detecting potyviruses have not targeted the most conserved sites. We are now testing primers that target the most highly conserved sites.

## Materials and Methods

### Datasets

An ‘all-sequence’ dataset obtained from the GenBank database in December 2005 contained 3484 potyvirus sequences including partial sequences. A ‘genome’ dataset was compiled that included 259 full length or near full length genomic sequences, greater than 9 kilobases in length, and representing 47 species. We aligned the genome dataset using the program MUSCLE [18] to produce an alignment that was 16017 nucleotides long. A ‘representative’ dataset was derived from the aligned genome dataset; it consisted of 47 sequences, one genome from each potyvirus species. We also assembled three ‘species’ datasets that consisted of all the complete aligned genome sequences from strains of the species *Turnip mosaic virus* (TuMV), *Potato virus Y* (PVY) *and Plum pox virus* (PPV); these datasets were of 61, 41 and 22 sequences respectively. These three species were chosen because they were represented in GenBank by the most genomic sequences.

Potyvirus species and sequences were counted using the all-sequence dataset and custom built software that sorted the dataset by name and date (‘Potyvirus-VirusBanker’, http://biojanus.anu.edu.au/programs/, 2006; Fourment M, Zheng, L and Gibbs, MJ). The number of potyvirus sequences obtained each year was determined by using the earliest date found in each GenBank sequence file. The number of potyvirus species recognised each year was determined by sorting the all-sequence dataset by date and by finding the earliest available sequence with a distinct species name. The earliest available date was assumed to be an estimate of the date when the species was confirmed to be distinct. We did not search the literature for earlier dates for the viruses and assumed that sequence comparisons were necessary for a species to be definitively distinguished. Our assumption about dates meant that species counts were made using the same timescale as sequence and nucleotide variant counts.

A procedure was developed to identify misnamed sequences and sequences from species that had not been officially recognised. Species names were obtained from the GenBank Definition annotation and these were cross-checked against the list of names from the International Committee on Taxonomy of Viruses (ICTV) [3]. Sequence records with names that were not listed by the ICTV were examined by searching for matching sequences in GenBank using the program BLASTN [19]. If a sequence with an unrecognised name was more than 85% identical to one of a known species, then it was assumed to belong to the species. If a sequence with an unrecognised name did not match one from a known species then it was considered to come from an “unrecognised species”. The taxonomic status of each sequence was also tested by cluster analysis. The conserved core regions of the coat protein gene sequences were extracted from the genome dataset alignment and used to calculate a neighbor-joining tree using the program MEGA3 [20]; the core regions were those bounded by the sequences encoding the WCIEN and QMKAAA motifs. The core regions were sorted into clusters according to their pair-wise nucleotide sequence identity using the program GROUPER (unpublished software, 2004; Armstrong, JS and Gibbs, AJ) with a clustering cut off point of 85% identity. Misnamed sequences found in the tree or the clusters were then tested by sequence matching using BLASTN [19]. Finally, the GenBank database was searched for nucleotide sequences with the names of the tentative potyvirus species listed by the ICTV [3] and the likely taxonomic status of those sequences was tested again following the procedure used for the unrecognised sequences.

### Conserved Sites

Conserved sites were named after the position of their 5′-most nucleotide in the aligned genome dataset. The sites were found in the aligned genome dataset using the program NCSF (unpublished software, http://biojanus.anu.edu.au/programs/, 2006; Wayper, P and Gibbs, MJ) and by eye using BioEdit [21]. Sites found by eye were chosen to be at least 15 nucleotides long and contain fewer than 3 consecutive variable bases and at least 10 nucleotides that were strictly conserved in more than 97% of sequences. NCSF was used with a search window 20 nucleotides long and four measures of conservation: Shannon informational entropy [22], redundancy [23], ‘average dominant base count’ and ‘sub-sequence variant counts’ . Average dominant base counts were calculated by counting the number of occurrences of the most common base at each position in the site and averaging those counts across all positions in the site. Sub-sequence variant counts were the counts of all the actual sequence variants seen at a site. The top five most conserved sites found using each measure and all of the sites found by eye were included in the analysis. Previously published conserved sites were found in the aligned genome dataset using BioEdit. As a control, 20 ‘random sites’ were selected by generating random numbers to define the 5′ position of sites 20 nucleotides long in the genome dataset alignment; sites that contained gaps were discarded.

Nucleotide variants (single nucleotide polymorphisms) were counted within each of the conserved and random sites. Genome sequences were ordered by the year in which they were submitted to the database, and then nucleotide variants were counted. Counts were made for each year using only the sequences available for that year. Counts were made for the years 1988 to 2005. The genome sequence of *Tobacco etch virus* (Accession code M11458) was used as the reference sequence as it was the first potyvirus genome publicly available in GenBank. Only those variants that had not been submitted in earlier years were counted so that identical nucleotides were not counted twice. Hence for each nucleotide position in a primer site, there were only three possible variants of the initial sequence, and a scale of 0 to 3 was used; invariant positions scored zero. Variant counts were summed across the whole site and the total divided by the length of the site to produce an average nucleotide variant count (N score). This calculation allowed sites of different lengths to be compared.

## Supporting Information

Table S1 Average nucleotide variant counts (N scores) of 17 conserved sites in representative potyvirus genomes from 1985 to 2005
(0.10 MB DOC)

Figure S1 The accumulated number of potyviruses species identified by their nucleotide sequences in GenBank from 1985 to 2005.
(3.89 MB TIF)

Figure S2 A yearly count of potyvirus species confirmed to be distinct by the deposition of a nucleotide sequence in GenBank from 1985 to 2005.
(3.46 MB TIF)

Figure S3 Average N score per sequence calculated by dividing the mean of N scores for all conserved sites by the number of sequences.
(2.70 MB TIF)
