# Supplementary material for: Accumulating Variation at Conserved Sites in Potyvirus Genomes Is Driven by Species Discovery and Affects Degenerate Primer Design
Source: PLoS One. 2008 Feb 13;3(2):e1586. doi: 10.1371/journal.pone.0001586 (PMC2217591; doi:10.1371/journal.pone.0001586)
Supplement: Table S1 — Average nucleotide variant counts (N scores) of 17 conserved sites in representative potyvirus genomes from 1985 to 2005 (0.10 MB DOC) [file pone.0001586.s001.doc]

TABLE S1 Average nucleotide variant counts (N scores) of 17 conserved sites in representative potyvirus genomes from 1985 to 2005

| **conserved sites** | **1985** | **1986** | **1987** | **1988** | **1989** | **1990** | **1991** | **1992** | **1993** | **1994** | **1995** | **1996** | **1997** | **1998** | **1999** | **2000** | **2001** | **2002** | **2003** | **2004** | **2005** |
| --- | --- | --- | --- | --- | --- | --- | --- | --- | --- | --- | --- | --- | --- | --- | --- | --- | --- | --- | --- | --- | --- |
| **7587** | 0.00 | 0.00 | 0.00 | 0.20 | 0.20 | 0.20 | 0.20 | 0.30 | 0.35 | 0.40 | 0.40 | 0.40 | 0.45 | 0.45 | 0.45 | 0.45 | 0.45 | 0.45 | 0.45 | 0.45 | 0.45 |
| **4545** | 0.00 | 0.00 | 0.00 | 0.10 | 0.25 | 0.25 | 0.30 | 0.45 | 0.45 | 0.50 | 0.50 | 0.50 | 0.50 | 0.50 | 0.50 | 0.50 | 0.50 | 0.50 | 0.50 | 0.50 | 0.50 |
| **9237** | 0.00 | 0.00 | 0.00 | 0.25 | 0.35 | 0.35 | 0.40 | 0.50 | 0.50 | 0.50 | 0.55 | 0.60 | 0.60 | 0.60 | 0.60 | 0.60 | 0.60 | 0.60 | 0.60 | 0.60 | 0.60 |
| **4539** | 0.00 | 0.00 | 0.00 | 0.09 | 0.26 | 0.26 | 0.30 | 0.43 | 0.43 | 0.43 | 0.52 | 0.52 | 0.52 | 0.57 | 0.57 | 0.61 | 0.61 | 0.61 | 0.61 | 0.61 | 0.61 |
| **7899** | 0.00 | 0.00 | 0.00 | 0.20 | 0.30 | 0.30 | 0.35 | 0.45 | 0.45 | 0.45 | 0.50 | 0.50 | 0.50 | 0.50 | 0.65 | 0.65 | 0.65 | 0.70 | 0.70 | 0.70 | 0.70 |
| **9162** | 0.00 | 0.00 | 0.00 | 0.18 | 0.29 | 0.29 | 0.29 | 0.35 | 0.35 | 0.47 | 0.53 | 0.53 | 0.53 | 0.53 | 0.53 | 0.53 | 0.53 | 0.59 | 0.65 | 0.65 | 0.71 |
| **8278** | 0.00 | 0.00 | 0.00 | 0.26 | 0.42 | 0.42 | 0.47 | 0.53 | 0.68 | 0.68 | 0.68 | 0.68 | 0.68 | 0.68 | 0.68 | 0.68 | 0.68 | 0.68 | 0.68 | 0.74 | 0.74 |
| **8853** | 0.00 | 0.00 | 0.00 | 0.10 | 0.25 | 0.25 | 0.25 | 0.45 | 0.45 | 0.50 | 0.55 | 0.65 | 0.65 | 0.65 | 0.65 | 0.65 | 0.65 | 0.70 | 0.70 | 0.70 | 0.80 |
| **4458** | 0.00 | 0.00 | 0.00 | 0.10 | 0.35 | 0.35 | 0.45 | 0.60 | 0.75 | 0.75 | 0.75 | 0.75 | 0.80 | 0.80 | 0.80 | 0.85 | 0.85 | 0.85 | 0.85 | 0.85 | 0.85 |
| **9099** | 0.00 | 0.00 | 0.00 | 0.15 | 0.30 | 0.30 | 0.35 | 0.45 | 0.45 | 0.45 | 0.60 | 0.65 | 0.75 | 0.80 | 0.85 | 0.85 | 0.85 | 0.85 | 0.85 | 0.85 | 0.85 |
| **7911** | 0.00 | 0.00 | 0.00 | 0.18 | 0.36 | 0.36 | 0.41 | 0.64 | 0.68 | 0.68 | 0.86 | 0.86 | 0.86 | 0.86 | 0.86 | 0.86 | 0.86 | 0.86 | 0.86 | 0.86 | 0.86 |
| **7722** | 0.00 | 0.00 | 0.00 | 0.25 | 0.30 | 0.30 | 0.40 | 0.45 | 0.50 | 0.50 | 0.55 | 0.65 | 0.70 | 0.70 | 0.80 | 0.85 | 0.85 | 0.85 | 0.85 | 0.90 | 0.90 |
| **4396** | 0.00 | 0.00 | 0.00 | 0.11 | 0.26 | 0.26 | 0.32 | 0.47 | 0.47 | 0.53 | 0.58 | 0.63 | 0.63 | 0.68 | 0.68 | 0.68 | 0.68 | 0.68 | 0.74 | 0.84 | 0.95 |
| **7545** | 0.00 | 0.00 | 0.00 | 0.17 | 0.30 | 0.30 | 0.35 | 0.48 | 0.65 | 0.70 | 0.74 | 0.83 | 0.87 | 0.91 | 0.91 | 0.96 | 0.96 | 0.96 | 0.96 | 0.96 | 0.96 |
| **3888** | 0.00 | 0.00 | 0.00 | 0.15 | 0.40 | 0.40 | 0.50 | 0.65 | 0.70 | 0.70 | 0.75 | 0.85 | 0.90 | 0.90 | 0.90 | 0.90 | 0.90 | 0.95 | 0.95 | 0.95 | 1.00 |
| **9042** | 0.00 | 0.00 | 0.00 | 0.20 | 0.30 | 0.30 | 0.55 | 0.70 | 0.70 | 0.80 | 0.80 | 0.85 | 0.90 | 1.00 | 1.00 | 1.00 | 1.00 | 1.10 | 1.10 | 1.10 | 1.10 |
| **8901** | 0.00 | 0.00 | 0.00 | 0.10 | 0.20 | 0.20 | 0.40 | 0.50 | 0.65 | 0.65 | 0.80 | 0.80 | 0.85 | 0.85 | 0.85 | 0.85 | 0.85 | 1.15 | 1.20 | 1.20 | 1.20 |
|  |  |  |  |  |  |  |  |  |  |  |  |  |  |  |  |  |  |  |  |  |  |
| **Mean** | **0.00** | **0.00** | **0.00** | **0.16** | **0.30** | **0.30** | **0.37** | **0.49** | **0.54** | **0.57** | **0.63** | **0.66** | **0.69** | **0.71** | **0.72** | **0.73** | **0.73** | **0.77** | **0.78** | **0.79** | **0.81** |
