# Supplementary figures and images for: Accumulating Variation at Conserved Sites in Potyvirus Genomes Is Driven by Species Discovery and Affects Degenerate Primer Design
Source: PLoS One. 2008 Feb 13;3(2):e1586. doi: 10.1371/journal.pone.0001586 (PMC2217591; doi:10.1371/journal.pone.0001586)

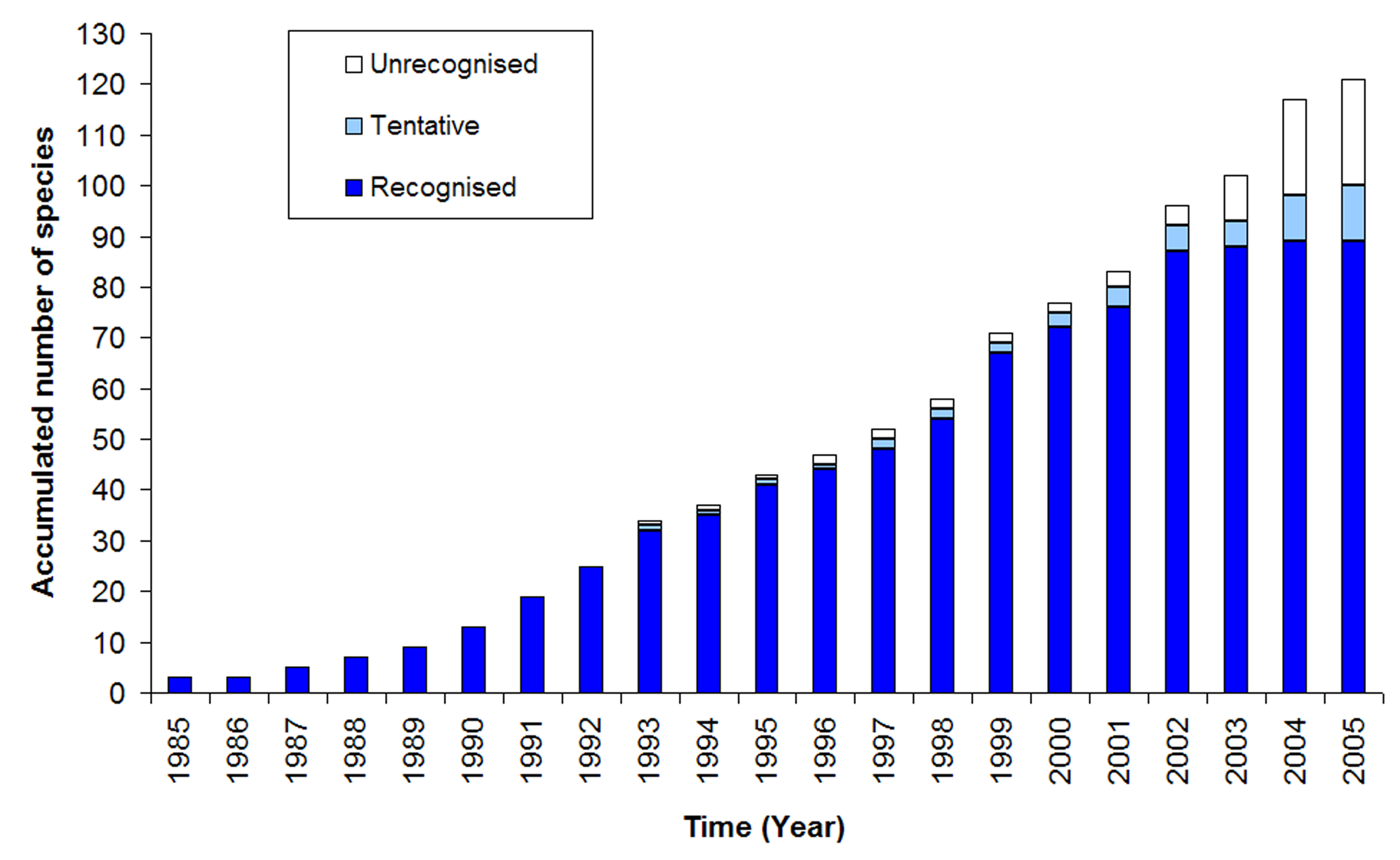

Supplement: Figure S1 — The accumulated number of potyviruses species identified by their nucleotide sequences in GenBank from 1985 to 2005. (3.89 MB TIF) [file pone.0001586.s002.tif]

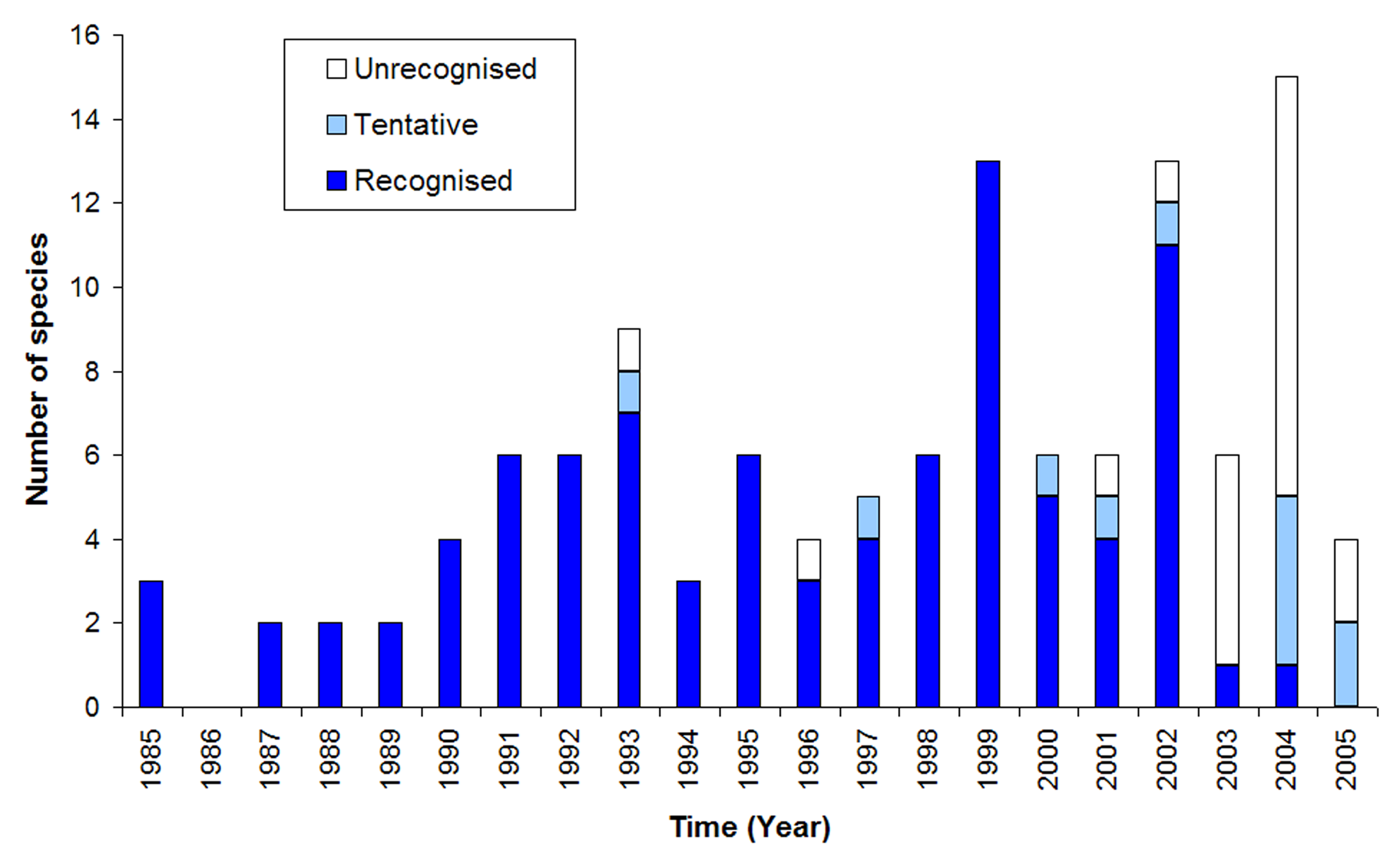

Supplement: Figure S2 — A yearly count of potyvirus species confirmed to be distinct by the deposition of a nucleotide sequence in GenBank from 1985 to 2005. (3.46 MB TIF) [file pone.0001586.s003.tif]

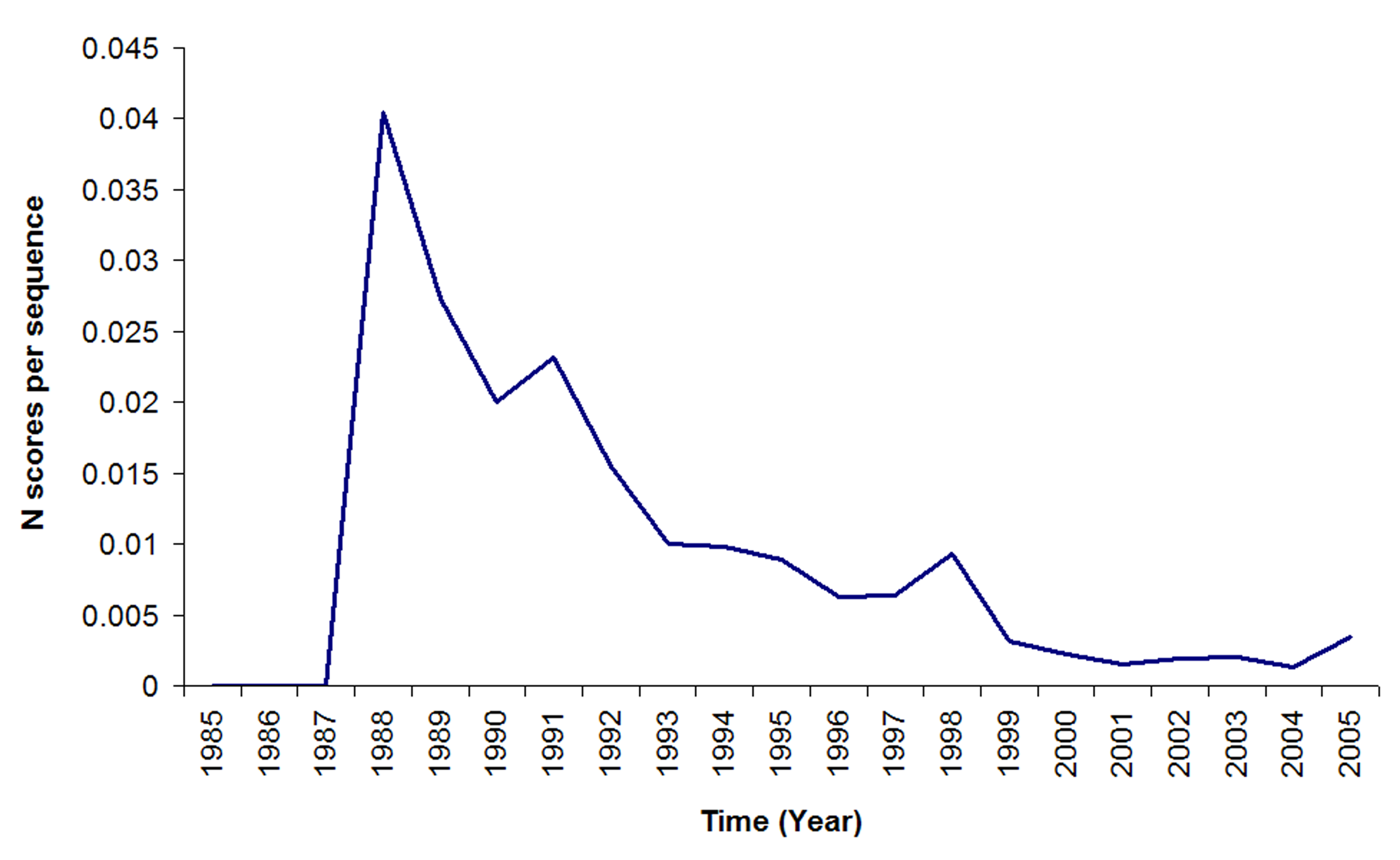

Supplement: Figure S3 — Average N score per sequence calculated by dividing the mean of N scores for all conserved sites by the number of sequences. (2.70 MB TIF) [file pone.0001586.s004.tif]
